# A New Physically Meaningful Threshold of Sample Entropy for Detecting Cardiovascular Diseases

**DOI:** 10.3390/e21090830

**Published:** 2019-08-25

**Authors:** Jinle Xiong, Xueyu Liang, Tingting Zhu, Lina Zhao, Jianqing Li, Chengyu Liu

**Affiliations:** 1 The State Key Laboratory of Bioelectronics, School of Instrument Science and Engineering, Southeast University, Nanjing 210096, China; 2 Department of Engineering Science, University of Oxford, Oxford OX3 7DQ, UK

**Keywords:** atrial fibrillation, cardiovascular time series, congestive heart failure, heart rate variability, sample entropy

## Abstract

Sample Entropy (SampEn) is a popular method for assessing the regularity of physiological signals. Prior to the entropy calculation, certain common parameters need to be initialized: Embedding dimension *m*, tolerance threshold *r* and time series length *N*. Nevertheless, the determination of these parameters is usually based on expert experience. Improper assignments of these parameters tend to bring invalid values, inconsistency and low statistical significance in entropy calculation. In this study, we proposed a new tolerance threshold with physical meaning ($r_{p}$), which was based on the sampling resolution of physiological signals. Statistical significance, percentage of invalid entropy values and ROC curve were used to evaluate the proposed $r_{p}$ against the traditional threshold ($r_{t}$). Normal sinus rhythm (NSR), congestive heart failure (CHF) as well as atrial fibrillation (AF) RR interval recordings from Physionet were used as the test data. The results demonstrated that the proposed $r_{p}$ had better stability than $r_{t}$, hence more adaptive to detect cardiovascular diseases of CHF and AF.

## 1. Introduction

Entropy provides valuable tools for quantifying the regularity of physiological time series and important insights to understand the basic mechanisms of the cardiovascular system. In order to better handle short time series in physiological signals, Pincus proposed approximate entropy (ApEn) when studying heart rate changes in sudden death in infants [1]. Since then, ApEn is widely used in many research fields [2,3]. However, due to the introduction of self-matching in the calculation process, ApEn contains estimated bias [4]. To solve the shortcomings of bias and relative inconsistency, Richman and Moorman developed sample entropy (SampEn), which was an improvement of ApEn and solved the problem of self-matching [4]. For evaluating the non-linear complexity in shorter time series, particular in physiological signals, SampEn is more adaptive compared to ApEn.

One of typical applications of SampEn in clinical measurement lies in distinguishing congestive heart failure (CHF) from normal sinus rhythm (NSR) [5,6]. As mentioned earlier, three common parameters such as embedding dimension *m*, tolerance threshold *r* and time series length *N* need to be initialized. However, it reveals several changes in clinical application: (1) Different values of tolerance threshold *r* lead to the inconsistency entropy results for CHF determination [7], (2) higher embedding dimension *m* might cause invalid entropy results in calculation, and (3) decrease in RR segment length is at the cost of lower statistical significance [8]. Thus, improving the performance of SampEn for physiological signal analysis has become an important issue.

For clinical applications, recommended *r* in ApEn is usually between 0.10 and 0.25 times the standard deviation (SD) of the physiological data [9]. Since SampEn is the improvement version of ApEn [4], these recommended parameter values are carried on as before [10,11]. Nevertheless, under certain circumstances, NSR group presented higher SampEn results than those in the CHF group when *r* was set to 0.10, while the outcomes reversed as *r* increased to 0.25 [8]. The inverted entropy results make it hard to establish a unified standard to detect CHF subjects with a constant *r* value. Therefore, our study proposes a solution to tackle the problem, employing a new mechanism to define threshold *r* to avoid the inconsistency of SampEn in CHF detection.

The growth of embedding dimension from *m* = 1 to *m* = 4 also witnessed a shrink in valid SampEn values for analyzing the typical 5 min RR time series [12]. Invalid entropy values appeared at higher embedding dimensions should be eliminated. As well, in most cases, invalid outcomes resulted from the division of similar vectors and dissimilar vectors. If the tolerance threshold was set too small, all vectors would overrun that boundary, thus they were regarded as dissimilar, leading to invalid SampEn results. The increase of *m* could only make the situation worse by expanding the distance between two vectors. Again, the reason of this problem lies in the selection of *r* values.

Besides detecting CHF subjects, SampEn also applies to atrial fibrillation (AF) detection [13]. Similar problems appear when recommended threshold is used to discriminate AF subjects. However, previous research has revealed that the constant threshold (*r* = 30 ms) performed better than the commonly used threshold (*r* = 0.20) when analyzing short-time AF segments [14]. This finding enlightened us to explore thresholds with physical meaning in SampEn calculation. We hypothesized the analogous conclusion would also apply to AF detection.

This study aims to examine whether threshold with physical meaning would be better than traditionally recommended threshold during SampEn calculation. Verifications will be performed on CHF and NSR groups, AF and non-AF groups, to validate the effect of physically meaningful threshold. The rest of paper is organized as follows. Section 2 describes the algorithm of SampEn and its limitation in clinical application. On that basis, the proposed threshold is introduced. The experiment process and results are presented in Section 3 and Section 4 respectively. Section 5 concludes the study.

## 2. Methods

### 2.1. Sample Entropy

SampEn was taken as a baseline algorithm in this study. The calculation process of SampEn was summarized as follows [4,15]:

For RR segment *x*(*i*) derived from a recording with length *N*, where 1 ≤ *I* ≤ *N*, given the parameters *m* and *r*, the vector sequences $X_{I}^{m}$ can be formulated as:

(1) $$X_{i}^{m}=\left\{x\left(i\right),\;x\left(i+1\right),\cdots ,x\left(i+m-1\right)\right\}\;1\leq i\leq N-m$$

The vector $X_{i}^{m}$ represents *m* consecutive *x*(*i*) values. Then the distance between $X_{i}^{m}$ and $X_{j}^{m}$ based on the maximum absolute difference is defined as:

(2) $$d_{i,j}^{m}=d\left[X_{i}^{m},X_{j}^{m}\right]=\underset{0\leq k\leq m-1}{\max}\left|x\left(i+k\right)-x\left(j+k\right)\right|$$

For each $X_{i}^{m}$, we denote $B_{i}^{m}$(*r*) as (*N* − *m*)^−1^ times the number of $X_{j}^{m}$ (1 ≤ *j* ≤ *N* − m) that meets $d_{i,\;j}^{m}\leq r$. Similarly, we set $A_{i}^{m}$(*r*) as (*N* − *m*)^−1^ times the number of $X_{j}^{m+1}$ that meets $d_{i,\;j}^{m+1}\leq r$ for all 1 ≤ *j* ≤ *N* − *m*.

Then SampEn is defined by

(3) $$SampEn=\left(m,\;r\;,\;N\right)=-ln\left(\sum_{i=1}^{N-m}A_{i}^{m}\left(r\right)/\sum_{i=1}^{N-m}B_{i}^{m}\left(r\right)\right)$$

Herein, we pre-define two parameters in the calculation of entropy metrics: Embedding dimension *m* = 1, 2, 3, 4 and tolerance threshold *r* = 0.10, 0.15, 0.20 and 0.25 times the standard deviation of the RR interval time series. Since the appropriate embedding dimension *m* is suggested to deal with the time series with a length of 10*^m^* to 10*^m^*^+1^, a relatively large *m* may lead to inefficient entropy results, thus we use *m* no more than 4. Likewise, the values of *r* we choose are verified to provide stable outputs for certain RR interval time series. The length of time series usually varies largely, from dozens such as 75 points, to up to thousands of points. Meanwhile, time series that contains less than 200 points is not recommended for either ApEn or SampEn because of inadequate vector matching [16,17]. We therefore select the time series length *N* to be 300 and 1000 to check the influence of various-size RR interval segments [18].

### 2.2. How Vector Similarity Changes When r Changes

Typically, recommended *r* for clinical use is between 0.10 and 0.25 times the standard deviation (SD) of the data. A greater SD will increase the determination threshold for consideration of a vector matching and vice versa with a smaller SD [1,17]. Studies have also proved that choosing a higher *r* value of 0.25 or 0.3 then the relationship becomes unstable with respect to changing data length [19]. Conversely, choosing a smaller *r* can lead to an increased number of self-matches [20]. Moreover, SampEn has been suggested to be highly dependent on signal-to-noise ratio [19,21]. To avoid a significant noise contribution on SampEn computation, one must choose *r* larger than most of the noise. Hence, the selection of *r* appears to be the most difficult to choose. When *r* is determined, there will be a vector distance distribution matrix consist of 0 and 1 for the time series [22]. However, there might be no changes in the corresponding distance matrix when *r* varies from 0.10 to 0.25. This motivated our work in exploring the nature of the problem.

**Figure 1:**
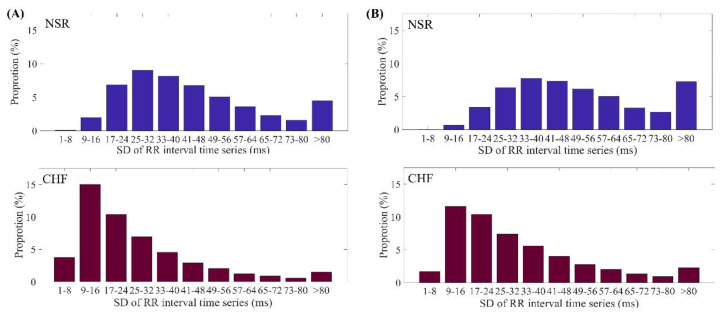
Distribution ranges of SD values of RR intervals for NSR and CHF groups when (**A**) *N* = 300 and (**B**) *N* = 1000.

Herein we take the CHF analysis for demonstration. As physiological signals were sampled at a specific frequency, the sampling resolution played a key role in the time series. ECG signals of both NSR and CHF groups were digitized at 128 Hz [23], which means the interval between every two sampling point is approximately 8 ms. Thus, to make *r* larger than the sampling resolution under recommended values, the SD of time series should be from 32 to 80 ms. In fact, most RR intervals of ECG signals can’t reach this range [24]. Figure 1 presented the SD distribution of RR intervals from NSR group and CHF group when *N* = 300 and 1000 respectively. Considering the sampling resolution of ECG signals was 128 Hz, SD below 32 ms was invalid. Nevertheless, for *N* = 300 in NSR group, SD under 32 ms was nearly 40% of all RR intervals from 54 subjects. The same result was approximately 75% of all RR intervals from 29 subjects in CHF group, even worse than the NSR group. When *N* was extended to 1000, though less obvious, the same outcome was observed, where 25% of NSR group and 50% of CHF group had SD under 32 ms, respectively.

**Figure 2:**
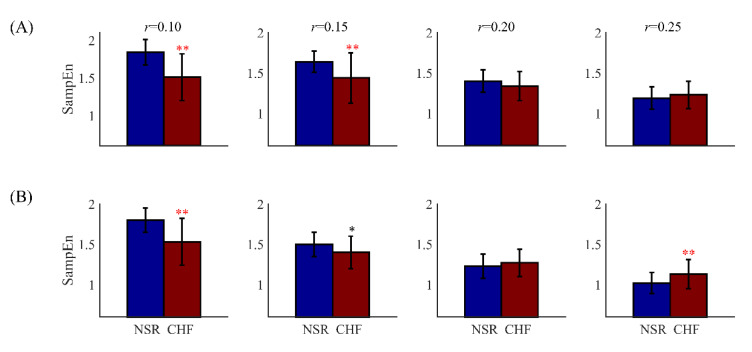
The distribution ranges of SampEn between NSR and CHF groups at different setting of tolerance threshold when (**A**) *N* = 300, *m* = 2 and (**B**) *N* = 1000, *m* = 2. The symbol ‘*’means statistical significance *p* < 0.05 and ‘**’means statistical significance *p* < 0.01, using *t*-test.

In previous research, the inconsistency of SampEn was reported for distinguishing CHF from NSR subjects [8]. The problem was showed in Figure 2 by box plot. As *r* increased from 0.10 to 0.25, the SampEn values of NSR group were first higher than those of the CHF group, then became lower. Therefore, it was hard to distinguish CHF from NSR as there existed no regularity for the relation between entropy values from these two different groups.

Based on these shortcomings of current SampEn, a new threshold method of *r* needs to be explored. Combining the analysis of RR intervals, we proposed a new tolerance threshold named as $r_{p}$, which has physical meaning over sampling resolution. We denoted the traditional tolerance threshold as $r_{t}$ hereafter. The details of these two thresholds are summarized below.

### 2.3. Selection of r Value: Traditional or Physically Meaningful

**Figure 3:**
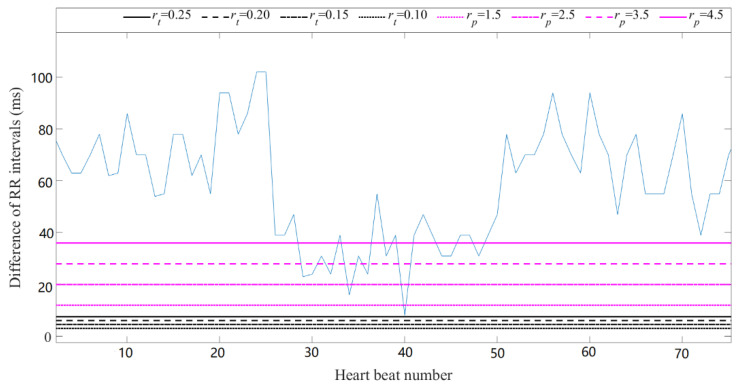
Presentation of traditional thresholds and physically meaningful thresholds in time difference of RR intervals in a CHF subject.

When the physically meaningful *r* was applied to time series, the direct and effective relation between RR intervals and threshold was presented. This is shown in Figure 3 using a CHF subject as an example. The $r_{t}$ values were 0.10, 0.15, 0.20 and 0.25, and $r_{p}$ values were 12 ms, 20 ms, 28 ms and 36 ms, which were presented as a cut point of sampling period (8 ms) in the legend. As shown in Figure 3, for most of time, $r_{t}$ was mostly below the minimum time difference of RR intervals, explaining why SampEn did not change over various $r_{t}$. However, the magenta lines of $r_{p}$ intersected the curve of RR interval time difference more frequently, leading to the significant entropy variance as $r_{p}$ changed.

**Figure 4:**
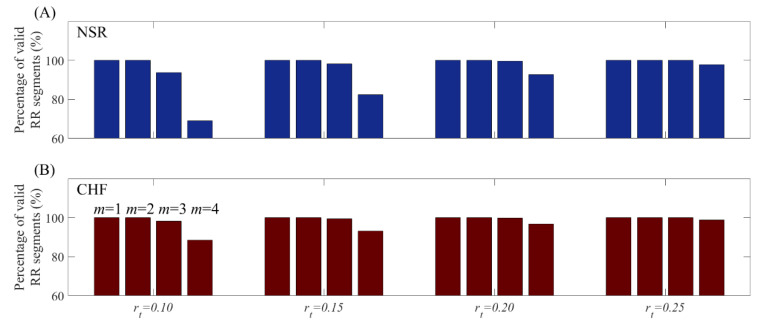
Percentage of valid RR segments at *m* = 1, 2, 3 and 4 respectively when using $r_{t}$ for (A) NSR group and (B) CHF group. The length of RR time series is 300.

When the product of threshold and SD is smaller than most time difference of RR intervals, it leads to two outcomes. If the product is larger than a sampling period, the SampEn value is valid, otherwise it is not. Since time series with slight heart rate variation is common in clinical data, changing $r_{t}$ value makes SampEn results unpredictable. Figure 4 shows the percentage of valid RR segments at *m* = 1, 2, 3 and 4 combined with $r_{t}$ from 0.10 to 0.25 under *N* = 300. Although for *m* = 1 and 2, all RR segments presented valid entropy results, the increase of *m* might lead to invalid values at certain proportion for both NSR and CHF groups. In contrast, $r_{p}$ is directly determined by multiples of sampling period, which possesses certain matching degree to the time series, thus avoids invalid values in entropy calculation fundamentally.

### 2.4. New Calculate Method for SampEn

Thus, a new calculate method for SampEn was proposed based on the conception of $r_{p}$. When processing a time series, we use its sampling resolution to calculate the corresponding sampling period. The physically meaningful threshold then is determined as non-integer multiples of sampling period, which can be either integer or non-integer, but has to be larger than one sampling period. Once $r_{p}$ has been determined, the same algorithm of SampEn is applied according to Equations (1) to (3).

First, entropy measures the conditional probability that two short vectors of length *m* that match within a distance tolerance $r_{p}$ will also match at the *m* + 1 st point. Thus, the determination for vector similarity is crucial, which relays on the measure of the distance between two vectors. Chebyshev distance (i.e., the element maximum distance) is applied here according to the traditional usage [13]. Second, once we have the distances between the two vectors, we can determine their similarity or dissimilarity using a determination rule function. In the definition of SampEn, similarity of vectors is based on Heaviside function [4,10]. The main feature of the Heaviside function is that it provides a step function that converts the input into activity equal to 0 or 1. It leads to a kind of conventional two-state classifier, where an input pattern is judged its belongingness to a given class by whether it satisfies certain precise properties required of membership [25]. The contributions of all the data points inside the boundary are treated equally, while the data points just outside the boundary are left out. Third, a probability-based estimation is used to generate the entropy value.

## 3. Data and Experiment

### 3.1. Data

Variations of RR intervals could be described by the conventionally accepted term “heart rate variability” (HRV), which analyzes the interval between consecutive beats [26]. Since HRV was confirmed to be a strong and independent predictor of mortality after an acute myocardial infarction, clinical importance has been attached to it. With the availability of new ECG recorders such as Holter, HRV has the potential to provide additional valuable insight into physiological and pathological conditions. For example, the analysis of HRV can give insight into autonomic abnormalities, which is an important aspect of heart failure [27]. This could also explain why heart failure subjects represent reduced HRV. Moreover, HRV is also a hallmark of AF. Study has found that HRV was greater in patients with lone AF than in those with cardiac disorders [28]. Therefore, in this study, we chose inter-beat interval time series data on both heart failure and AF subjects.

Two MIT-BIH RR interval time series databases were used from http://www.physionet.org [23], a free-access, on-line archive of physiological signals. The NSR RR Interval Database was used as the non-pathological and control group data. This database included 54 long-term RR interval recordings of subjects in normal sinus rhythm aged 29 to 76. The CHF RR Interval Database was used as the pathological group data. This database included 29 long-term RR interval recordings of subjects aged 34 to 79, with congestive heart failure (NYHA classes I, II, and III). Each of the long-term RR interval recordings is 24 h long including both day-time and night-time. Both the NSR and CHF subjects took the Holter ECG measurement under the similar level of physical activity. The original ECG signals were digitized at 128 Hz, and the beat annotations were obtained by automated analysis with a manual review and correction.

MIT-BIH AF database and MIT-BIH arrhythmia database were used to test the AF RR interval time series data. The MIT-BIH AF database includes 25 long-term ECG recordings with rhythm and beat annotation files. Individual ECG recordings are 10 h in duration and were sampled at 250 Hz, resulting in a minimum temporal resolution of 4 ms for the RR time series. Rhythm annotations were performed manually for four types: AF, AFL (atrial flutter), J (AV junctional rhythm) and N (used to indicate all other rhythms). Beat annotations were prepared using an automated detector with two recordings (no. 05091 and no. 07859) corrected manually. The MIT-BIH arrhythmia database includes 48 short-term (30 min) ECG recordings. This database includes 23 subjects with non-AF rhythms and eight AF subjects with both AF rhythm and a variety of non-AF rhythms. The sampling rate was 360 Hz, giving a minimum temporal resolution of about 3 ms for the RR time series. Beats were annotated independently by at least two cardiologists. The NSR RR Interval Database mentioned above was also used as the non-pathological and control group data in AF analysis.

### 3.2. Experiment Scheme

**Figure 5:**
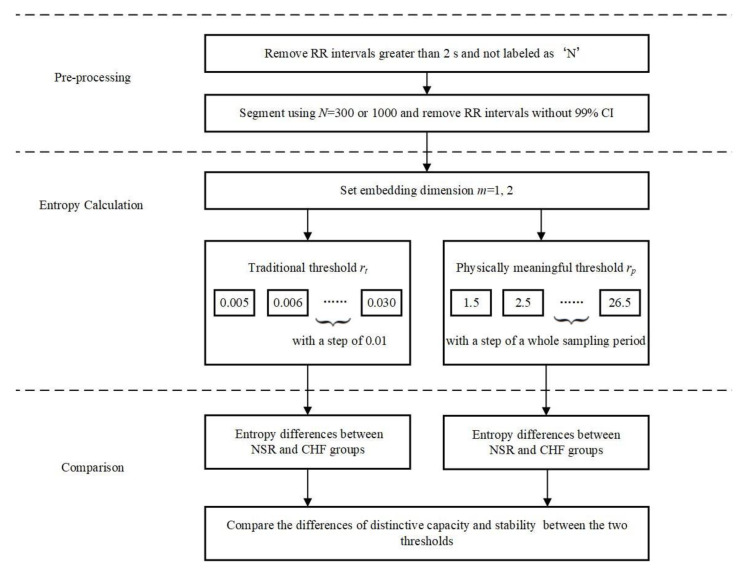
Block diagram of the proposed evaluation process for CHF analysis. NSR: normal sinus rhythm, CHF: congestive heart failure, CI: confidence interval.

Figure 5 shows the block diagram of the evaluation process for CHF detection used in this study. It consists of three major steps. Equation (1) pre-processing and segmenting for each RR interval recording; Equation (2) entropy calculation for each RR segment with different combinations of parameters; and Equation (3) comparison between NSR and CHF groups to determine whether SampEn with physically meaningful threshold is better than the traditional SampEn.

In Equation (1), the RR intervals greater than 2 s were first removed from the raw RR interval recordings to ignore the influence from the artifacts. For each beat in the raw ECG signals, it was annotated as a normal (denoted as ‘N’) or abnormal heartbeat. The abnormal heartbeats were usually caused by the ectopic beats such as supra-ventricular ectopic beats or ventricular ectopic beats, depending on the localization of the ectopic focus. The RR intervals derived from the abnormal heartbeats could confound the entropy analysis of HRV [29], and therefore were removed from the RR interval recordings. Table 1 shows the total number of RR intervals for both NSR and CHF groups, as well as the numbers of RR intervals after these two removing procedures. After that, we used two different length windows *N* to segment the long-term RR interval recordings to form the RR segments for the entropy calculation. In this study, we set *N* = 300 and *N* = 1000 respectively to observe the performances of entropy measures for different length of RR segments. We did not consider the overlapping operation between adjacent N-length windows since the previous study reported that overlapping between adjacent *N*-length windows did not improve atrial fibrillation organization estimation with respect to the analysis of non-overlapping windows [30]. Table 1 also shows the total numbers of RR segments for both NSR and CHF groups when setting *N* = 300 and *N* = 1000, respectively. For each RR segment, we removed the RR intervals without 99% confidence interval (CI), (i.e., ± 3 × SD).

In Equation (2), SampEn with different thresholds were used to calculate the entropy values for each RR segment under the different parameter settings: embedding dimension *m* was set as 1 and 2 respectively, and $r_{t}$ was set from 0.05 to 0.30 with a step of 0.01 for SampEn. We further set $r_{p}$ from 1.5 times to 26.5 times sampling period with a step of one sampling period, reasoning that threshold within a sampling period makes no difference to results. As the original ECG signals were digitized at 128 Hz, we considered the sampling period to be 8 ms approximately.

In Equation (3), the entropy results were compared between the NSR and CHF groups under the different combinations of parameters *m*, *r* and *N*, aiming to explore whether $r_{p}$ is superior to $r_{t}$ in distinguish the CHF patients from the NSR subjects.

**Figure 6:**
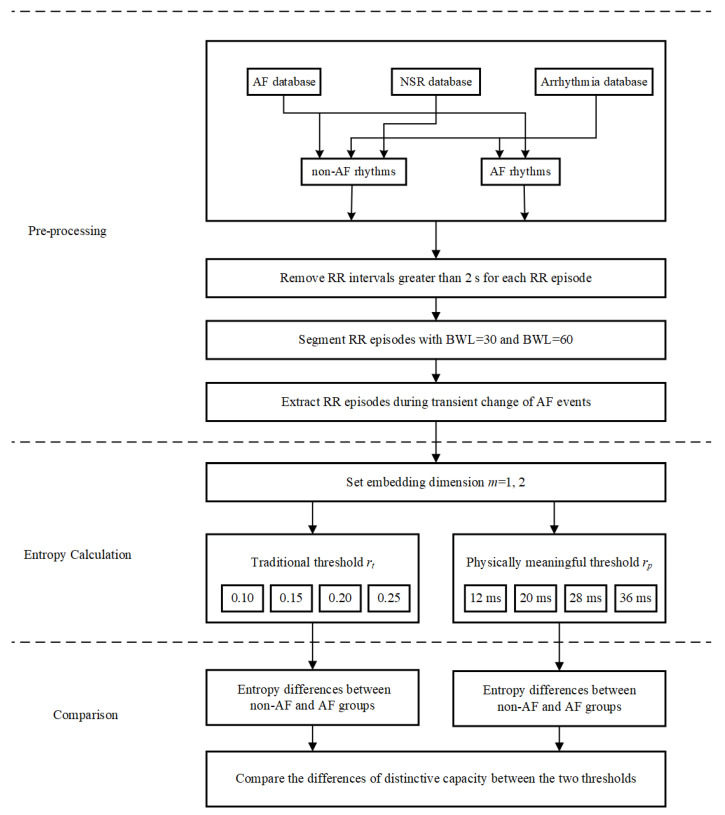
Block diagram of the proposed evaluation process for AF analysis. AF: atrial fibrillation, NSR: normal sinus rhythm, BWL: beat window length.

Figure 6 shows the block diagram of the evaluation process for AF analysis used in this study. Likewise, it also consists of three major steps. Equation (1) pre-processing and segmenting for each RR interval recording; Equation (2) entropy calculation for each RR segment with different combinations of parameters; and (3) comparison between non-AF and AF groups to determine whether SampEn with physically meaningful threshold is better than the traditional SampEn.

In Equation (1), for the MIT-BIH AF database, arrhythmia database and NSR database, all RR time series were regarded as either non-AF rhythm or AF rhythm. Data pre-processing was performed on the classified RR episodes. RR intervals greater than 2 s were removed to eliminate the influence of the missed QRS detection due to noise or ECG electrode drop out. Two types of beat window length (BWL)—30 and 60 beats—were used to segment RR episodes without overlap. Table 2 shows the detailed database profile.

In Equation (2), embedding dimension *m* was set as 1 and 2, respectively. As for traditional threshold, we still used 0.10, 0.15, 0.20 and 0.25 for AF subjects and the control group. Nevertheless, the sampling resolutions for the MIT-BIH AF database and MIT-BIH arrhythmia database were different from the NSR RR Interval Database, thus the set of physically meaningful threshold needed to be considered carefully. Noticing the lowest sampling frequency of these databases was 128 Hz, we supposed the sampling period for all the ECG signals was still 8 ms. Thus, we chose to adopt the previous $r_{p}$ values 1.5, 2.5, 3.5 and 4.5 times sampling period 8ms for AF analysis, which were 12 ms, 20 ms, 28 ms and 36 ms in time domain. Since the sampling frequency for the MIT-BIH AF database and MIT-BIH arrhythmia database were 250 Hz and 360 Hz respectively, such set of $r_{p}$ could meet our demand.

In Equation (3), the entropy results were compared between the non-AF and AF groups under the different combinations of parameters *m*, *r* and *BWL*. These entropy results were compared between the AF and non-AF rhythm types.

### 3.3. Statistical Analysis

When applying to CHF detection, for each RR segment length of *N* = 300 and *N* = 1000, there were 52 entropy values for each RR segment using SampEn with $r_{t}$ (two embedding dimensions and 26 traditional thresholds). Likewise, there were also 52 entropy values for each RR segments using SampEn with $r_{p}$ (two embedding dimensions and 26 physically meaningful thresholds). The overall mean and SD values of these two methods were calculated across all RR interval recordings, separately for the NSR and CHF groups. Student’s *t*-test was used to test the statistical difference between the two groups. All statistical analyses were performed using the MATLAB software (Version R2017a, The MathWorks, Natick, USA). Statistical significance was reported with *p* < 0.05. To prove that the proposed $r_{p}$ is also reliable for time series with other segment length, we added statistical tests at *N* = 5000 and *N* = 10,000 to verify its effectiveness.

Furthermore, the receiving operator curve (ROC) curve and the index of area under the curve (AUC) were used to evaluate the effectiveness of SampEn using different thresholds in CHF detection. Entropy values on one side of a threshold *c* were labelled as CHF while values on the other side of *c* were labelled as NSR. Classifier accuracy was assessed via the following performance metrics:

- Sensitivity: *Se* = TP/(TP+FN)
- Specificity: *Sp* = TN/(TN+FP)

where TP, TN, FP and FN are the numbers of true positives, true negatives, false positives and false negatives respectively. The ROC curve is a plot (*Se*) versus (1-*Sp*) for many possible values of *c*, which varied from the minimum to the maximum of the entropy outputs, with a step of 1% of the range.

Unlike the analysis of CHF subjects with time series measuring hundreds of RR intervals, entropy calculation related to AF subjects uses short time series [31]. In this study, we used AF episodes with BWL of 30 and 60 beats to compare the performances of $r_{t}$ and $r_{p}$. Similarly, after the calculation of entropy values, student’s t-test was used to test the statistical difference between the non-AF and AF groups. The proportion of invalid values was listed out as well. 

### 3.4. Stability Test

In clinical applications, signals are commonly contaminated by artefacts, such as a drift and interference caused by several bioelectric phenomena, or by intrinsic noise from the recorder or noise from electrode-skin contact [32]. If a turbulence could cause SampEn to change dramatically, the determination to distinguish CHF subjects from NSR subjects might lead to a wrong diagnosis. Thus, we tested the robustness of SampEn for both traditional threshold and physically meaningful threshold and compared them to determine whether the proposed threshold had better stability.

## 4. Results

### 4.1. Results of CHF & NSR

**Figure 7:**
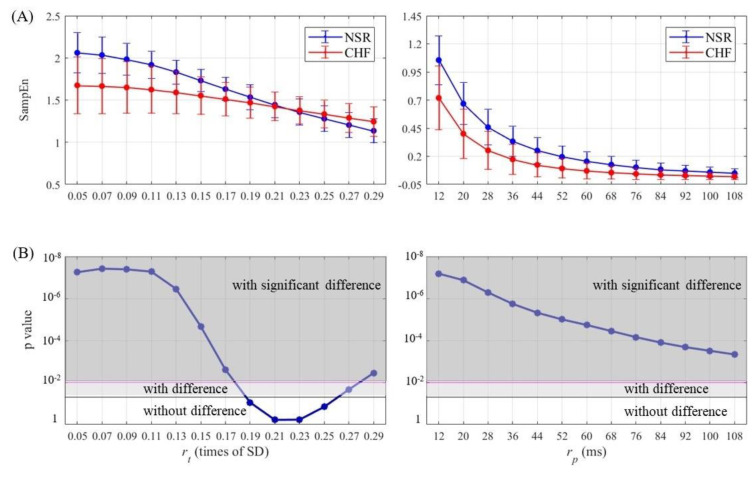
Entropy calculation and statistical analysis contrasting $r_{t}$ (left) with $r_{p}$ (right) between NSR and CHF groups at *N* = 300 and *m* = 1 for (**A**) SampEn results and (**B**) statistical significance.

**Figure 8:**
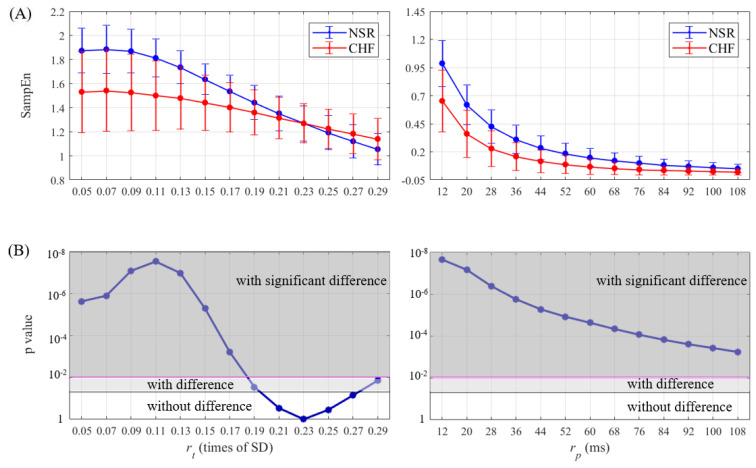
Entropy calculation and statistical analysis contrasting $r_{t}$ (left) with $r_{p}$ (right) between NSR and CHF groups at *N* = 300 and *m* = 2 for (**A**) SampEn results and (**B**) statistical significance.

**Figure 9:**
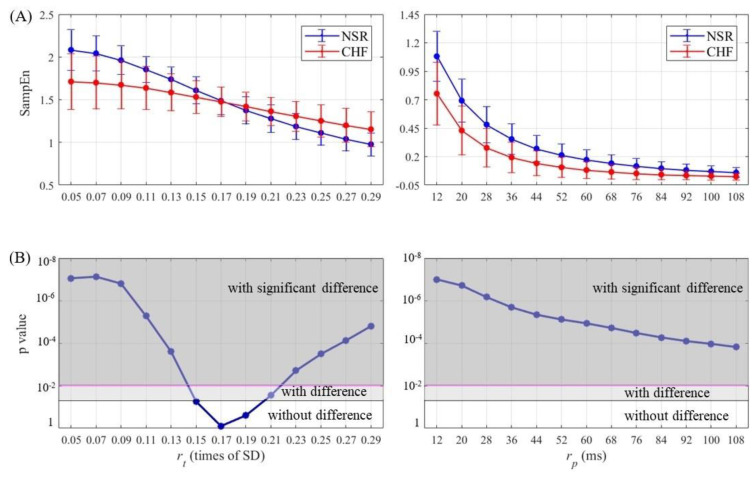
Entropy calculation and statistical analysis contrasting $r_{t}$ (left) with $r_{p}$ (right) between NSR and CHF groups at *N* = 1000 and *m* = 1 for (**A**) SampEn results and (**B**) statistical significance.

**Figure 10:**
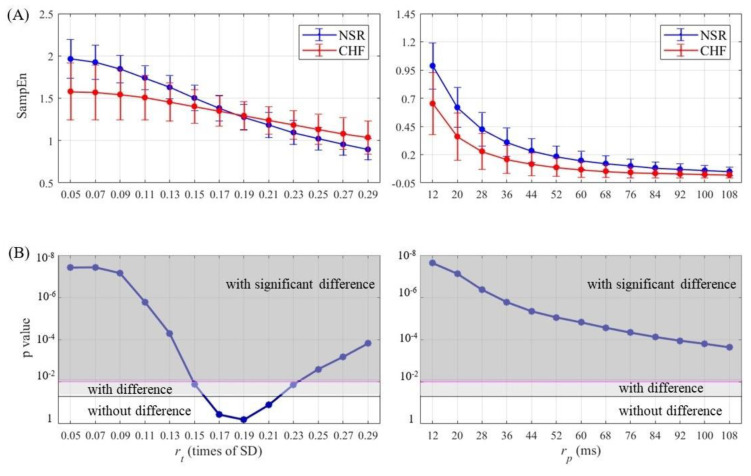
Entropy calculation and statistical analysis contrasting $r_{t}$ (left) with $r_{p}$ (right) between NSR and CHF groups at *N* = 1000 and *m* = 2 for (**A**) SampEn results and (**B**) statistical significance.

SampEn results as well as statistical significance were calculated and then plotted for half of entropy values listed in Section 3 in Figure 7, Figure 8, Figure 9 and Figure 10. The lengths of RR segments were 300 and 1000, and embedding dimension was set as 1 and 2, respectively. For SampEn with traditional threshold of all combinations of (*N*, *m*), the blue line of NSR and the red line of CHF intersected at a particular point in the plot, and negative logarithm of *p* value first decreased but then increased as $r_{t}$ increased, revealing the inconsistency when using $r_{t}$. In contrast, the lines of two different groups remained separate for SampEn with physically meaningful threshold, as negative logarithm of *p* value monotonically decreased. In fact, when converting $r_{t}$ to time period by multiplying SD, the traditional threshold only equaled to a relatively small part at the beginning of the $r_{p}$ curve, thus the *p* value was non-monotonic. Moreover, the minimum value of negative logarithm of *p* value for $r_{p}$ was still above the magenta line (*p* value = 0.01). Therefore, the use of $r_{p}$ in SampEn performed better in detecting CHF. Besides, smaller $r_{p}$ values such as 1.5 times sampling period (i.e., 12 ms) turned out to be more statistically significant.

Table 3 shows results of SampEn with $r_{t}$ or $r_{p}$ for the two groups using different combinations of (*m*, *r*) when setting *N* = 300 and *N* = 1000. Since the traditional threshold values 0.10, 0.15, 0.20 and 0.25 are commonly used, we selected these four values for $r_{t}$ in Table 3, and the most statistically significant $r_{p}$ values (12, 20, 28, 36 ms, i.e., 1.5, 2.5, 3.5 and 4.5 times sampling period) for the proposed method. As shown in Table 3, for *N* = 300, SampEn with $r_{t}$ had statistical significances only for *m* = 1 and 2 combined with *r* = 0.10 and 0.15. However, SampEn with $r_{p}$ had statistical significances for all thresholds when *m* = 1 and 2. When extending RR segment length to *N* = 1000, SampEn with $r_{t}$ had statistical significances for combinations satisfying *r* = 0.10 and 0.25 as well as *m* = 2 combined with *r* = 0.15. In comparison, SampEn with $r_{p}$ remained almost the same amount of statistical significances as those for *N* = 300. The proportion of the combinations of (*m*, *r*) to statistically distinguish the two groups out of all calculated combinations for traditional SampEn was 50% when *N* = 300 and 62.5% when *N* = 1000. In comparison, the same proportion was 100% at both *N* = 300 and *N* = 1000 for the proposed method. Thus, SampEn with physically meaningful threshold might be more adaptive to shorter time series when detecting CHF. In addition, it is important to note that traditional SampEn values in the NSR group were larger than those in the CHF group when *r* = 0.10, 0.15 and 0.20 but lower when *r =* 0.25, implying no consistency existed between NSR and CHF groups. By contrast, SampEn values from the proposed method in the NSR group were consistently higher than those in the CHF group. Therefore, the inconsistency of traditional SampEn was solved by the use of our new proposed physically meaningful threshold method.

Besides the commonly used values *N* = 300 and *N* = 1000, similar calculation was performed on *N* = 5000 and *N* = 10,000 to explore the statistical significance. Table 3 also shows the entropy values as well as *p* values for NSR and CHF groups when RR segment length was extended dramatically with different (*m*, *r*) combinations. The results prove that reliability of SampEn using $r_{p}$ for time series data of 5000 and 10,000 samples still exists. Although nearly all of the parameter combinations using $r_{t}$ have statistical significance, their *p* values are larger than those using $r_{p}$. Thus, our proposed threshold presents better distinctive capacity over time series data of different length.

**Figure 11:**
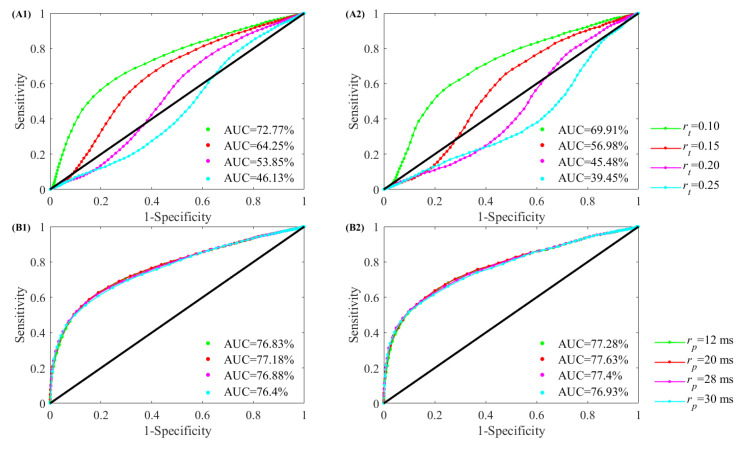
ROC curve plots with AUC values for the four values of $r_{t}$ and $r_{p}$ in the RR Interval Databases for classifying NSR and CHF subjects. The top two sub-figures (**A1**–**A2**) show SampEn results using $r_{t}$, while bottom sub-figures (**B1**–**B2**) show SampEn results using $r_{p}$. Different combinations of *m* and *N* were used: (**A1**) and (**B1**) for *m* = 1 and *N* = 300, and (**A2**) and (**B2**) for *m* = 1 and *N* = 1000.

Figure 11 illustrates the ROC curves with AUC values obtained using different thresholds for classifier testing. To classify NSR and CHF subjects for each parameter combination, $r_{p}$ = 20 ms, $r_{p}$ = 28 ms, $r_{p}$ = 12 ms and $r_{p}$ = 36 ms resulted in the highest to lowest AUCs, in order. For *m* = 1 and *N* = 300, the AUC values were 77.18%, 76.88%, 76.83% and 76.40% respectively for the four thresholds, and for *m* = 1 and *N* = 1000, the AUC values were 77.63%, 77.40%, 77.28% and 76.93% respectively. Meanwhile, for traditional threshold, $r_{t}$ = 0.10, $r_{t}$ = 0.15, $r_{t}$ = 0.20 and $r_{t}$ = 0.25 resulted in the highest to lowest AUCs, in order. For *m* = 1 and *N* = 300, the AUC values were 72.77%, 64.25%, 53.85% and 46.13% respectively for the four thresholds, and for *m* = 1 and *N* = 1000, the AUC values were 69.91%, 56.98%, 45.48% and 39.45% respectively. All AUCs using $r_{p}$ were higher than those using $r_{t}$. These results reveal that the entropy calculation with $r_{p}$ is superior to the use of $r_{t}$. Moreover, the relation between AUC value and the selection of *m* and *N* seems to be obscured.

### 4.2. Results of AF & Non-AF

**Figure 12:**
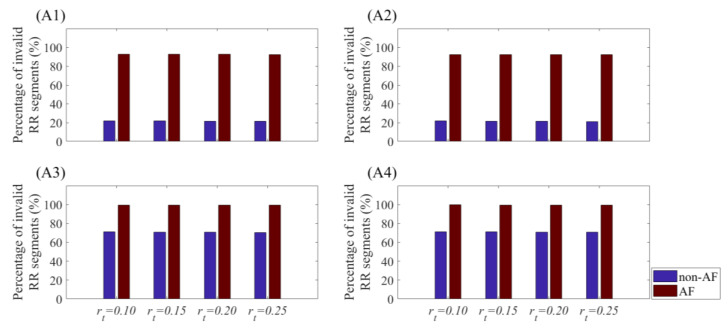
Percentage of invalid RR segments for the four values of $r_{t}$ in the RR Interval Databases for classifying AF and non-AF subjects. Different combinations of *m* and BWL were used: (**A1**) For *m* = 1 and BWL = 30, (**A2**) *m* = 1 and BWL = 60, (**A3**) for *m* = 2 and BWL = 30, and (**A4**) for *m* = 2 and BWL = 60.

To further examine the efficiency of $r_{p}$ on AF detection, analogous calculation was performed. Figure 12 shows the percentage of invalid RR segments for classifying AF and non-AF subjects when $r_{t}$ was applied. When *m* = 1 and BWL = 30, for four different threshold values, the percentages of invalid values for non-AF group were around 22%, while those for AF group were about 93%. As embedding dimension *m* increased to 2 with the same BWL, the proportions of invalid values for non-AF group increased dramatically, which even exceeded 70%. Meanwhile, the corresponding percentages for AF group reached almost 100%. Moreover, when BWL was set as 60, the results were pretty much the same. Since there are too many invalid values, SampEn with traditional threshold would be improper in AF detection.

On the contrary, the calculation with $r_{p}$ turned out to be relative desirable. When setting *m* = 1, for both 30-beat and 60-beat data, no invalid entropy value existed. As *m* increased to 2, for both BWL = 30 and 60, the first two thresholds presented invalid values merely for AF group. The percentage of invalid RR segments was 10% for $r_{p}$ = 12 ms, and 0.5% for $r_{p}$ = 20 ms. Thus, the increase of embedding dimension caused mild influence to the SampEn calculation with $r_{p}$.

Table 4 shows the analysis results of these non-AF and AF data. When using $r_{t}$, the SampEn values of AF group were lower than those of non-AF group. Meanwhile, several parameter combinations did not have statistical significance. Since even $r_{p}$ = 12 ms was larger than $r_{t}$ = 0.25 when converted to time domain, the use of $r_{t}$ was actually not stable. Considering the various RR interval lengths of AF subjects, the corresponding SampEn values would be higher than the non-AF subjects. However, such trait was not presented when $r_{t}$ was applied. In contrast, when using $r_{p}$, the SampEn values of AF group were significantly higher than non-AF group, which was in accordance with the characteristic of AF subjects. The corresponding *p* values also implied that all parameter combinations have statistical significance at *p* < 0.01. Therefore, the superiority of $r_{p}$ over $r_{t}$ has been proved again.

### 4.3. Stability Analysis

In account of any unexpected artefacts in original ECG signals, we also compared the robustness between the usage of $r_{t}$ and $r_{p}$. According to the statistical significance pointed out by Figure 7, Figure 8, Figure 9 and Figure 10, the first four values (12, 20, 28 and 36 ms) taken as physically meaningful threshold had relative better statistical significance, thus we used these four thresholds to analyze the stability of $r_{p}$. Meanwhile, due to the widely use of *r* = 0.10, 0.15, 0.20 and 0.25 in clinical applications, we also took them as traditional thresholds to check their stability. The stability test was performed on both *N* = 300 and *N* = 1000 when *m* = 1.

**Figure 13:**
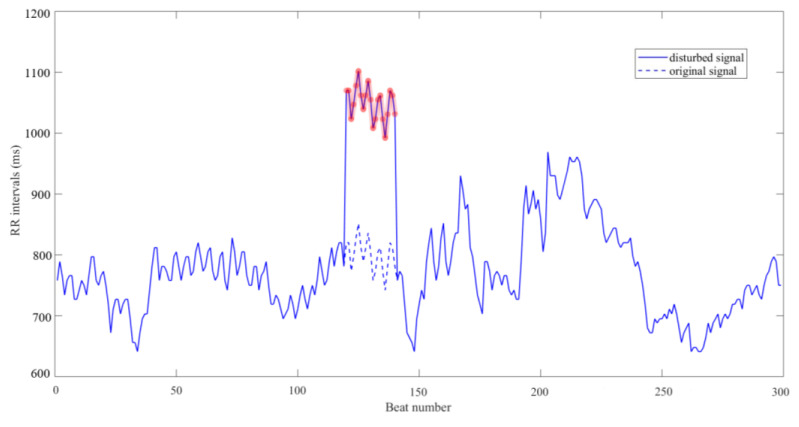
A DC drift that elongating 200 ms of RR interval length in 20 heart beats out of the original ECG signal for a CHF subject.

To simulate artefacts introduced in the original signals [33], we chose 20 consecutive heart beats out of each RR segment (*N* = 300 or 1000 respectively) randomly, and added them with an extra time period of 200 ms by introducing a DC drift, as shown in Figure 13. Then we calculated all RR segments from both 54 NSR subjects and 29 CHF subjects to obtain new SampEn values. The relative errors were then calculated according to the SampEn results without artefacts. Since four different values under $r_{t}$ and $r_{p}$ were analyzed, we compared the robustness when using two thresholds in form of bar plot.

In order to further explain the simulative artefacts added to ECG signals, we considered the signal from the first RR segment of the CHF subject numbered 201 as an example and calculated its SampEn at *N* = 300 and *m* = 1 using $r_{t}$ and $r_{p}$, respectively. In account of the ability to detect CHF in operation, $r_{t}$ was set as 0.15 and $r_{p}$ as 36 ms. Then we added 200 ms to its heart beats from number 121 to 140, and the whole RR segment containing 300 heart beats was showed in Figure 10. As the figure indicates, a DC drift was applied to the signal, thus the change in SampEn result of this RR segment might influence the average value of the subject. Under this situation, we calculated the SampEn values again and compared their variation. When using $r_{t}$, the original SampEn result was 0.0388 and the impacted one was 0.0517, which was a 33% increase of the previous value. Meanwhile, when $r_{p}$ was applied, the original SampEn result was 0.4719 and the drifted one was 0.4635, indicating the latter decreased only 2% when compared to the former. When more subjects from the database were tested, the same outcome that $r_{p}$ changed at a lower rate still appeared.

Change percentage when DC drifts were enforced on ECG signals from the different combinations of *m*, *r* and *N* was presented in Table 5. When *N* = 300, for all four threshold values, $r_{p}$ presented smaller change percentage than $r_{t}$, which implied our proposed physically meaningful threshold was more stable when facing sudden drifts. The growth of *m* also showed the increase of change percentages for both NSR and CHF groups. The same result appeared at *N* = 1000. When threshold increased, the change percentage increased as well, but $r_{p}$ increased at a lower rate than $r_{t}$. The analysis under both circumstances confirmed $r_{p}$ had better robustness than $r_{t}$. Moreover, when an extra period of time was subtracted from one heartbeat interval, the same conclusion still applied.

## 5. Discussion

As the change of tolerance threshold sometimes generated the same result in traditional SampEn calculation, this study turned to the ECG signal itself and explored the relation between tolerance threshold and sampling resolution. Aiming at the shortcoming of SampEn in AF detection, researchers have changed the selection method for threshold parameters [14]. One process in their study involved comparison between variable threshold and constant threshold, then the threshold was determined to obtain a minimum numerator count of 5. Later examination with short-time AF episodes proved that the use of 30 ms as a constant threshold would be more stable than the traditional threshold *r* = 0.20. Our conception of adjusting SampEn in CHF detection partly came from this research. To avoid the inconsistency and invalid values in previous method [8], we proposed a new tolerance threshold with physical meaning, and verified its superiority over the traditional threshold, $r_{t}$. Actually, the examinations on both heart failure and AF data verified that constant threshold with physical meaning would be more effective.

To test the clinical validity of the novel threshold $r_{p}$, 83 subjects were enrolled (54 normal subjects and 29 heart failure patients). SampEn with various combinations of (*N*, *m*) and statistical differences for both $r_{t}$ and $r_{p}$ were analyzed. The consistency of SampEn results and statistical significance for $r_{p}$ revealed it had a better performance in detecting CHF subjects compared to $r_{t}$. The advantages of the proposed $r_{p}$ are: (1) It avoided the invalid entropy values in each RR segment, (2) the selection of $r_{p}$ was determined by the sampling resolution of physiological signals, thus more stable when applied to real clinical applications, and (3) the flexible $r_{p}$ presented better robustness when dealing with fluctuation in signals.

As mentioned above, when using $r_{t}$, the increase of embedding dimension *m* led to the increase of invalid entropy values for RR segments, thus the mean SampEn for one subject might not exist [34]. Moreover, the decrease of RR segment length *N* made the situation even worse. In contrast, our proposed $r_{p}$ was taken sampling resolution into account, and subsequently avoided the invalid entropy values in calculation. Its adaptability to shorter time series made it more proper for clinical applications.

In this study, the original ECG signals for NSR and CHF groups were digitized at 128 Hz, but the product of $r_{t}$ multiplying standard deviation might be smaller than one sampling period. Although raising sampling resolution would solve the problem, it is expensive and not practical. Since different physiological signals have their own sampling resolution and $r_{p}$ was represented in the form of sampling period multiples, the variation of tested signals has no effect on the final outcome. The stability of $r_{p}$ overcame the defects in using traditional threshold when facing various ECG signals. Considering the fluctuation in original ECG signals caused by unexpected reasons, we also compared the robustness between the usage of $r_{t}$ and $r_{p}$. The results proved that $r_{p}$ was less vulnerable to the sudden fluctuation of ECG signals than $r_{t}$, therefore it has better robustness. When encountering turbulence in practical applications, the proposed threshold $r_{p}$ showed a lower change rate, thus the discrimination for CHF subjects would remain stable.

Some error factors, such as the magnitude of signals and the amount of noise when collecting signals, would alter analysis process, thus lead to different results. The outcomes of our experiments point out that these factors probably cause the performance issues of traditional SampEn method. Since the selection of traditional threshold is prone to be affected by the noise, the higher entropy values of NSR groups turn into the opposite results as threshold increases, which demonstrates such instability. However, the instability is improved by the proposed physically meaningful threshold, and noise analysis in our study has proved this.

There are limitations in this study. First, although we considered traditional threshold from 0.05 to 0.30 with a step of 0.01, and physically meaningful threshold from 1.5 to 26.5 times of sampling period with a step of a whole sampling period for our general analysis, the calculation of statistical significance and robustness was only performed on some threshold values. Those in-between values still remained uncertain. Second, larger embedding dimension *m* was not considered in the current study, and in account of clinical applications, it would be more favorable to test the proposed $r_{p}$ under shorter RR segment length *N*. Third, to further explore the advantage of $r_{p}$ over the traditional threshold, more analysis such as sensitivity and specialty should also be estimated. Moreover, the superiority of $r_{p}$ over the traditional threshold should be tested across multiple databases.

In conclusion, the current study has put forward a new physically meaningful threshold based on the sampling resolution of ECG signals for SampEn in detecting cardiovascular diseases. The better performance of the proposed threshold $r_{p}$ over traditional threshold $r_{t}$ was proved in the analysis of statistical significance and stability. Our proposed threshold also avoided the invalid entropy results during the traditional SampEn calculation and could be applied according to the sampling period or sampling resolution of ECG signals. Therefore, the proposed $r_{p}$ would be more adaptive and stable in clinical applications and has better performance in cardiovascular diseases detection.

## Figures and Tables

**Table 1:** Statistical results of the numbers of RR interval recordings, RR intervals and RR segments from the 54 NSR and 29 CHF RR Interval Databases.

Variables	NSR Group	CHF Group
Name of RR interval recordings	nsr001–nsr054	chf201–chf229
No. of RR interval recordings	54	29
No. of RR intervals	5,790,504	3,312,195
No. of RR intervals after removing greater than 2 s	5,780,148	3,306,394
No. of RR intervals after removing abnormal heartbeats	5,738,937	3,102,120
No. of RR segments when setting *N* = 300	19,101	10,324
No. of RR segments when setting *N* = 1000	5711	3089

**Table 2:** Statistical results of the data profile for AF and non-AF rhythms from the MIT-BIH NSR database, MIT-BIH AF database and MIT-BIH arrhythmia database.

Variable	AF Rhythm	Non-AF Rhythm
No. of rhythm episodes	406 (16.9%)	1999 (83.1%)
No. of RR intervals	533,085 (8.3%)	5,892,134 (91.7%)
No. of RR intervals after removing greater than 2 s	533,029 (7.5%)	6,529,842 (92.5%)
No. of RR segments (30-beat)	17,591 (7.4%)	218,798 (92.6%)
No. of RR segments (60-beat)	8709 (7.4%)	109,215 (92.6%)

**Table 3:** SampEn from the different combinations of embedding dimension *m* changed from 1 to 2 and tolerance threshold *r* when setting segment length *N* = 300 and *N* = 1000. The traditional $r_{t}$ changed from 0.10 to 0.25 with a step of 0.05 and physically meaningful $r_{p}$ changed from 12 ms to 36 ms (i.e., 1.5 times sampling period to 4.5 times sampling period) with a step of one sampling period 8 ms. *P*-value indicates the statistical significance between the NSR and CHF groups at each combination of (*m*, *r*). Data are expressed as number or mean ± standard deviation (SD). ‘*’: statistical significance *p* < 0.05, ‘**’: statistical significance *p* < 0.01.

Threshold Value	Group	*N* = 300		*N* = 1000		*N* = 5000		*N* = 10,000	
		*m* = 1	*m* = 2	*m* = 1	*m* = 2	*m* = 1	*m* = 2	*m* = 1	*m* = 2
**Traditional**									
$r_{t}$ = 0.10	NSR	1.95 ± 0.18	1.84 ± 0.17	1.91 ± 0.16	1.80 ± 0.15	1.76 ± 0.45	1.63 ± 0.46	1.66 ± 0.48	1.53 ± 0.49
	CHF	1.64 ± 0.30	1.51 ± 0.31	1.66 ± 0.27	1.53 ± 0.29	1.63 ± 0.34	1.49 ± 0.36	1.63 ± 0.33	1.48 ± 0.35
	*p*-value	4 × 10^−8^ **	7 × 10^−8^ **	7 × 10^−7^ **	3 × 10^−7^ **	5 × 10^−9^ **	1 × 10^−11^ **	0.32	0.08
$r_{t}$ = 0.15	NSR	1.73 ± 0.14	1.64 ± 0.13	1.61 ± 0.16	1.50 ± 0.15	1.33 ± 0.46	1.22 ± 0.45	1.18 ± 0.42	1.07 ± 0.42
	CHF	1.55 ± 0.23	1.44 ± 0.31	1.53 ± 0.19	1.40 ± 0.20	1.42 ± 0.46	1.28 ± 0.36	1.36 ± 0.35	1.22 ± 0.36
	*p*-value	2 × 10^−5^ **	5 × 10^−6^ **	0.055	0.013 *	2 × 10^−5^ **	6 × 10^−3^ **	1 × 10^−9^ **	1 × 10^−6^ **
$r_{t}$ = 0.20	NSR	1.49 ± 0.15	1.40 ± 0.14	1.33 ± 0.16	1.23 ± 0.15	1.05 ± 0.38	0.95 ± 0.38	0.95 ± 0.33	0.85 ± 0.33
	CHF	1.45 ± 0.18	1.34 ± 0.18	1.39 ± 0.17	1.27 ± 0.17	1.24 ± 0.38	1.10 ± 0.38	1.18 ± 0.37	1.04 ± 0.36
	*p*-value	0.26	0.10	0.091	0.31	8 × 10^−21^ **	7 × 10^−14^ **	3 × 10^−19^ **	2 × 10^−13^ **
$r_{t}$ = 0.25	NSR	1.28 ± 0.15	1.19 ± 0.14	1.11 ± 0.15	1.02 ± 0.13	0.87 ± 0.32	0.78 ± 0.32	0.78 ± 0.29	0.69 ± 0.29
	CHF	1.33 ± 0.17	1.23 ± 0.17	1.25 ± 0.19	1.13 ± 0.18	1.06 ± 0.39	0.93 ± 0.39	0.98 ± 0.39	0.85 ± 0.38
	*p*-value	0.14	0.35	3 × 10^−4^ **	0.003 **	2 × 10^−26^ **	1 × 10^−17^ **	2 × 10^−16^ **	3 × 10^−11^ **
**Physically Meaningful**									
$r_{p}$ = 12 ms	NSR	1.06 ± 0.22	0.97 ± 0.21	1.08 ± 0.22	0.99 ± 0.20	1.10 ± 0.33	0.99 ± 0.32	1.11 ± 0.32	0.99 ± 0.31
	CHF	0.72 ± 0.28	0.63 ± 0.28	0.75 ± 0.28	0.65 ± 0.28	0.77 ± 0.31	0.66 ± 0.32	0.79 ± 0.31	0.66 ± 0.31
	*p*-value	7 × 10^−8^ **	2 × 10^−8^ **	1 × 10^−7^ **	2 × 10^−8^ **	1 × 10^−76^ **	4 × 10^−84^ **	2 × 10^−38^ **	7 × 10^−42^ **
$r_{p}$ = 20 ms	NSR	0.67 ± 0.19	0.60 ± 0.17	0.69 ± 0.19	0.62 ± 0.18	0.71 ± 0.28	0.62 ± 0.27	0.72 ± 0.27	0.63 ± 0.26
	CHF	0.40 ± 0.22	0.34 ± 0.21	0.43 ± 0.22	0.36 ± 0.21	0.45 ± 0.25	0.36 ± 0.24	0.46 ± 0.24	0.37 ± 0.24
	*p*-value	1 × 10^−7^ **	7 × 10^−8^ **	7 × 10^−7^ **	8 × 10^−8^ **	7 × 10^−72^ **	1 × 10^−76^ **	2 × 10^−36^ **	1 × 10^−38^ **
$r_{p}$ = 28 ms	NSR	0.46 ± 0.16	0.41 ± 0.15	0.48 ± 0.16	0.42 ± 0.15	0.50 ± 0.23	0.43 ± 0.22	0.51 ± 0.23	0.43 ± 0.22
	CHF	0.25 ± 0.17	0.21 ± 0.16	0.28 ± 0.17	0.23 ± 0.16	0.30 ± 0.20	0.23 ± 0.19	0.31 ± 0.19	0.24 ± 0.19
	*p*-value	5 × 10^−7^ **	4 × 10^−7^ **	2 × 10^−6^ **	4 × 10^−7^ **	4 × 10^−65^ **	6 × 10^−67^ **	4 × 10^−33^ **	4 × 10^−34^ **
$r_{p}$ = 36 ms	NSR	0.33 ± 0.13	0.30 ± 0.12	0.35 ± 0.14	0.31 ± 0.13	0.37 ± 0.20	0.32 ± 0.19	0.38 ± 0.19	0.32 ± 0.18
	CHF	0.17 ± 0.13	0.15 ± 0.12	0.19 ± 0.13	0.16 ± 0.12	0.21 ± 0.16	0.17 ± 0.15	0.22 ± 0.16	0.17 ± 0.15
	*p*-value	2 × 10^−6^ **	2 × 10^−6^ **	5 × 10^−6^ **	2 × 10^−6^ **	1 × 10^−59^ **	3 × 10^−60^ **	1 × 10^−30^ **	5 × 10^−31^ **

**Table 4:** Entropy values and statistical significance of SampEn from the different combinations of embedding dimension *m* changed from 1 to 2 and tolerance threshold *r* when setting BWL = 30 and BWL = 60. The traditional $r_{t}$ changed from 0.10 to 0.25 with a step of 0.05 and physically meaningful $r_{p}$ changed from 12 ms to 36 ms (i.e., 1.5 to 4.5 times sampling period) with a step of one sampling period 8 ms. *P*-value indicates the statistical significance between the AF and non-AF groups at each combination of (*m*, *r*). Data are expressed as number or mean ± standard deviation (SD). ‘*’: statistical significance *p* < 0.05, ‘**’: statistical significance *p* < 0.01.

Threshold Value	Group	BWL30		BWL60	
		*m* = 1	*m* = 2	*m* = 1	*m* = 2
**Traditional**					
$r_{t}$ = 0.10	AF	2.01 ± 0.50	1.19 ± 0.48	2.03 ± 0.50	1.13 ± 0.50
	non-AF	2.24 ± 0.57	1.38 ± 0.48	2.24 ± 0.57	1.40 ± 0.49
	*p*-value	4 × 10^−8^ **	4 × 10^−8^ **	5 × 10^−9^ **	0.32
$r_{t}$ = 0.15	AF	2.01 ± 0.50	1.19 ± 0.49	2.03 ± 0.50	1.13 ± 0.49
	non-AF	2.24 ± 0.58	1.38 ± 0.48	2.23 ± 0.57	1.40 ± 0.49
	*p*-value	2 × 10^−5^ **	2 × 10^−5^ **	2 × 10^−5^ **	1 × 10^−9^ **
$r_{t}$ = 0.20	AF	2.01 ± 0.50	1.21 ± 0.49	2.03 ± 0.50	1.16 ± 0.49
	non-AF	2.24 ± 0.58	1.38 ± 0.48	2.23 ± 0.57	1.40 ± 0.49
	*p*-value	0.26	0.26	8 × 10^−21^ **	3 × 10^−19^ **
$r_{t}$ = 0.25	AF	2.02 ± 0.50	1.23 ± 0.50	2.04 ± 0.50	1.16 ± 0.50
	non-AF	2.23 ± 0.58	1.38 ± 0.48	2.23 ± 0.57	1.39 ± 0.49
	*p*-value	0.14	0.14	2 × 10^−26^ **	2 × 10^−16^ **
**Physically Meaningful**					
$r_{p}$ = 12 ms	AF	1.41 ± 0.49	1.34 ± 0.54	1.41 ± 0.33	1.34 ± 0.32
	non-AF	0.18 ± 0.25	0.72 ± 0.23	0.18 ± 0.31	0.16 ± 0.31
	*p*-value	7 × 10^−8^ **	7 × 10^−8^ **	1 × 10^−76^ **	2 × 10^−38^ **
$r_{p}$ = 20 ms	AF	0.98 ± 0.38	1.00 ± 0.45	0.98 ± 0.28	1.00 ± 0.27
	non-AF	0.11 ± 0.20	0.40 ± 0.18	0.11 ± 0.25	0.10 ± 0.24
	*p*-value	1 × 10^−7^ **	1 × 10^−7^ **	7 × 10^−72^ **	2 × 10^−36^ **
$r_{p}$ = 28 ms	AF	0.72 ± 0.32	0.46 ± 0.36	0.73 ± 0.23	0.73 ± 0.23
	non-AF	0.09 ± 0.17	0.25 ± 0.16	0.09 ± 0.20	0.08 ± 0.19
	*p*-value	5 × 10^−7^ **	5 × 10^−7^ **	4 × 10^−65^ **	4 × 10^−33^ **
$r_{p}$ = 36 ms	AF	0.55 ± 0.28	0.33 ± 0.30	0.55 ± 0.20	0.55 ± 0.19
	non-AF	0.08 ± 0.15	0.17 ± 0.15	0.07 ± 0.16	0.07 ± 0.16
	*p*-value	2 × 10^−6^ **	2 × 10^−6^ **	1 × 10^−59^ **	1 × 10^−30^ **

**Table 5:** Change percentage when impulses were enforced on ECG signals from the different combinations of embedding dimension *m* changed from 1 to 2 and tolerance threshold *r* when setting segment length *N* = 300 and *N* = 1000. The traditional $r_{t}$ changed from 0.10 to 0.25 with a step of 0.05 and physically meaningful $r_{p}$ changed from 12 ms to 36 ms with a step of one sampling period 8 ms.

Threshold Value	Group	*N* = 300		*N* = 1000	
		*m* = 1	*m* = 2	*m* = 1	*m* = 2
**Traditional**					
$r_{t}$ = 0.10	NSR	8.01% ± 3.11%	9.40% ± 3.23%	2.74% ± 1.22%	3.09% ± 1.36%
	CHF	4.19% ± 3.37%	4.88% ± 3.44%	1.38% ± 1.41%	1.49% ± 1.35%
$r_{t}$ = 0.15	NSR	34.16% ± 7.58%	35.34% ± 7.85%	9.15% ± 2.64%	9.46% ± 2.64%
	CHF	39.57% ± 9.13%	40.50% ± 11.72%	5.09% ± 3.89%	5.45% ± 4.06%
$r_{t}$= 0.20	NSR	32.62% ± 10.38%	33.79% ± 11.12%	14.10% ± 5.53%	14.80% ± 6.03%
	CHF	51.67% ± 15.50%	53.81% ± 16.09%	13.93% ± 8.00%	15.37% ± 8.57%
$r_{t}$ = 0.25	NSR	33.44% ± 10.25%	35.38% ± 11.92%	14.76% ± 6.97%	15.34% ± 7.07%
	CHF	52.84% ± 15.79%	54.81% ± 16.13%	28.79% ± 16.71%	30.29% ± 17.13%
**Physically Meaningful**					
$r_{p}$ = 12 ms	NSR	1.66% ± 0.19%	2.04% ± 0.25%	0.52% ± 0.07%	0.61% ± 0.09%
	CHF	2.46% ± 0.93%	2.73% ± 0.91%	0.72% ± 0.22%	0.83% ± 0.25%
$r_{p}$ = 20 ms	NSR	2.41% ± 0.49%	2.82% ± 0.55%	0.71% ± 0.11%	0.84% ± 0.16%
	CHF	5.78% ± 7.85%	6.31% ± 7.94%	1.33% ± 0.89%	1.55% ± 1.00%
$r_{p}$ = 28 ms	NSR	5.23% ± 9.20%	5.82% ± 9.70%	0.97% ± 0.20%	1.14% ± 0.25%
	CHF	17.69% ± 24.66%	18.77% ± 24.69%	1.88% ± 2.02%	2.11% ± 2.18%
$r_{p}$= 36 ms	NSR	8.68% ± 11.05%	9.64% ± 11.90%	1.57% ± 1.94%	1.81% ± 2.01%
	CHF	31.62% ± 28.61%	33.48% ± 29.46%	9.53% ± 16.70%	12.83% ± 22.59%
